# Mining the risk: early cardiovascular detection in workers

**DOI:** 10.3389/fmed.2025.1678172

**Published:** 2025-11-27

**Authors:** Ricardo Jorquera, Guillermo Droppelmann, Max Dollmann, Gonzalo Blanco, Ignacio Ahumada, Alfonso Lira, Felipe Feijoo

**Affiliations:** 1 Workmed, Santiago, Chile; 2 Blackmind-AI, Santiago, Chile; 3 School of Industrial Engineering, Pontificia Universidad Católica de Valparaíso, Valparaíso, Chile

**Keywords:** blood glucose, body mass index, cardiovascular risk, machine learning, occupational health

## Abstract

**Background:**

Cardiovascular disease (CVD) is the leading cause of death worldwide. Although tools exist to assess individual cardiovascular risk (CVR), they often fall short in unique populations such as miners, who work under extreme conditions. To address these limitations, this study proposes the use of machine learning (ML) and longitudinal data to predict risk progression using accessible clinical markers. Body mass index (BMI) and blood glucose (BG) were chosen as key CVR proxies because they are affordable, measured routinely in occupational health checks, and responsive to metabolic stresses common in mining environments.

**Methods:**

We conducted a retrospective longitudinal analysis of 89,045 Chilean mining workers (420,966 preemployment exams; 2021–2024). For each worker, we formed successive visit pairs to model transitions between clinically defined BMI and BG categories. Four binary outcomes based on the scenario per biomarker were specified (any upward transition; adjacent upward transition; obesity–morbid obesity/prediabetes–diabetes; any transition ending in morbid obesity/diabetes). Machine learning techniques were built to assess transitions for each scenario and biomarker. We applied a stratified 70/30 train–test split, repeated 7-fold cross-validation within training, random hyperparameter search (AUC objective), and downsampling of the majority classes within folds to address the imbalance. Performance in the original (imbalanced) test set was summarized by AUC, accuracy, sensitivity, and specificity with 95% CIs of the cross-validation process. The correlation between models was assessed using Pearson's correlations of predicted probabilities.

**Results:**

Predicting BMI transitions (*N* = 18,035 pairs) was highly accurate between models. The best performance occurred for severe progression (Scenario 4, defined as any transition ending in morbid obesity): where XGB achieved AUC 0.95 and accuracy 0.91, with high sensitivity and strong specificity. For broader BMI transitions across scenarios 1–3, models remained reliable AUC 0.84–0.87. BG transitions (*N* = 16,161 pairs) were harder but still actionable. The strongest results were for progression to diabetes (Scenario 4), with RF reaching AUC 0.83 (95% CI: 0.82–0.90) and accuracy 0.76; other BG scenarios yielded AUC 0.71–0.77. Cross-validation closely matched test performance. Pairwise probability correlations were typically >0.90 for BMI and >0.80 for BG in severe scenarios, indicating good generalization and no evidence of overfitting.

**Conclusion:**

ML models effectively predict clinically relevant BMI and BG risk transitions in the extraction of occupational health data. The use of longitudinal visit pairs and scenario-based evaluation improves the capacity of the models to achieve high AUC values and maintain accuracy and sensitivity, while ensuring generalization and consistency. These findings highlight the potential of this approach to improve the assessment of CVR and support preventive decision-making in high-risk working populations.

## 1 Introduction

Cardiovascular diseases (CVD) are responsible for more than 20.5 million deaths per year, accounting for more than a third of global mortality, currently representing the greatest mortality threat facing humanity (1). The impact is so significant that, in a single year, it exceeds all deaths recorded during the COVID-19 pandemic (2). CVD often begins silently and asymptomatically. However, it can rapidly progress to severe clinical manifestations such as ischemic heart disease, stroke, heart failure, and arrhythmias, leading to a substantial burden of morbidity and mortality. This underscores the critical need to implement effective early detection strategies (3, 4).

Current evidence suggests that an essential component of CVD prevention is the early identification of high-risk individuals, allowing timely interventions and reducing both the disease burden and its socioeconomic impact (5). Individual cardiovascular risk (CVR), reflects the probability of experiencing a major cardiovascular event over a given period of time, usually 5 or 10 years (6). This risk is determined by multiple factors, including body mass index (BMI) and blood glucose (BG), two widely available and routinely used clinical indicators, whose association with cardiovascular events is well established in the literature (7–9). Indeed, a BMI ≥ 35 is associated with a 43% increase in CVD risk in men and a 32% increase in women. Similarly, hyperglycemia in people with diabetes increases this risk by 75% in men and 87% in women, which contributes in particular to heart failure (10). Given the limitations in many occupational settings, there is a growing interest in simplifying CVR assessment by using routine low-cost biomarkers such as BMI and BG, especially in environments where traditional tools are impractical due to cost, logistics or lack of comprehensive clinical data (11, 12). Given their strong independent associations with cardiovascular events, BMI and BG are particularly well suited for longitudinal monitoring in occupational health programs (13). In fact, the Pan American Health Organization (PAHO) includes both BMI and BG as key elements for CVR evaluation and prevention strategies, particularly in resource-limited settings, as outlined in their guidelines for cardiovascular risk stratification (14).

The distribution, significance and evolution of these markers can differ significantly in populations exposed to demanding work conditions. In the mining population, this association was reported more than 30 years ago (15, 16). This group of workers is of particular interest in public and occupational health due to their prolonged exposure to harsh environments characterized by high physical workloads, long shifts, thermal stress, and, frequently, hypoxia from high-altitude work (17). These metabolic stress conditions contribute to an unfavorable CVR profile compared to the general population. This is strongly associated with high rates of hypertension, abdominal obesity, metabolic disorders, and sleep disturbances (18–20).

In Chile, mining is one of the main economic drivers of the country, employing more than 800,000 people in operations mainly located at high altitude in the northern and central regions (21, 22). Studies in the Chilean mining population have reported significant rates of metabolic syndrome and CVR, far exceeding those observed in the general population, reinforcing the need for targeted surveillance and predictive tools adapted to the occupational and individual context of these workers (17, 23). Despite this, there are several tools for CVR stratification, such as the Framingham equations and other scales proposed by the WHO or PREVENT, developed by the American Heart Association (10, 24, 25). The predictive power of these CVR models is well-established for estimating the 10-year risk of CVD events, with values of the area under the receiver operating characteristic curve (AUC-ROC) typically ranging from 0.70 to 0.82, depending on the model and population (26–28), although their predictive performance often varies depending on regional calibration and population-specific characteristics. The Framingham Risk Score, developed from a United States cohort, achieves AUC-ROC values of 0.75–0.78 for the prediction of coronary heart disease (26), while the SCORE model, designed for European populations, reports AUC-ROC values of 0.70–0.75, with performance varying by regional calibration (27). The PREVENT model, introduced by the American Heart Association, incorporates additional risk factors such as kidney function and social determinants, demonstrating an AUC-ROC of 0.82 for atherosclerotic CVD events, surpassing the 0.76 of the Pooled Cohort Equations in recent validations (10). However, these models may exhibit reduced capacity when applied to diverse populations due to variations in the prevalence of risk factors and event rates, often requiring recalibration to avoid over or underestimation of risk (29, 30). In addition, most risk scores do not incorporate the longitudinal evolution of clinical indicators such as BMI and BG, which are particularly relevant in occupational surveillance programs where periodic measurements are available. This underscores the need for population-specific validation to ensure robust risk stratification, particularly in unique settings like occupational health. In fact, these tools are widely used and were developed based on cohorts of the general population, with a limited representation of workers exposed to extreme conditions. This limits their external validity, reduces clinical applicability, and compromises their relevance in occupational health contexts. Furthermore, a systematic review of the Framingham rule reported that, among 40 studies that validated it in external populations, 67.5% found poor performance. However, the model was neither reconfigured nor updated in these cases (31).

For these reasons, there is an urgent need to develop alternative methods that optimize CVR assessment in mining workers, using easily available clinical indicators adapted to the unique characteristics of this population. In this study, rather than estimating CVR scores *per se*, we focus on predicting clinically relevant transitions in the BMI and BG categories over time as proxies of increasing CVR (11, 12). This transition-based approach has practical advantages: it facilitates interpretation by occupational physicians, enables early preventive actions, and aligns closely with regulatory thresholds used in worker health evaluations. This approach leverages routinely available and low-cost data while capturing meaningful changes in the risk profile of workers exposed to extreme occupational stressors (32). BMI and BG were selected as target variables because they are easy to obtain, inexpensive and are routinely measured in occupational health assessments. Both have been consistently associated with an increase in CVR and are sensitive to typical metabolic and environmental stressors of mining work, making them practical proxies to identify high-risk scenarios in this population (33).

In this context, machine learning (ML) has emerged as a promising tool to predict clinical events and improve diagnostic processes. ML models can identify complex patterns and nonlinear relationships between variables, often undetectable by traditional statistical methods (34). This ability is particularly relevant when analyzing large volumes of longitudinal data, such as those generated in periodic occupational health assessments, where subtle changes in indicators such as BMI and glucose over time may provide critical information to anticipate the progression of CVR (35). Additionally, ML models offer the potential to improve the consistency and reproducibility of evaluations, mitigating the intra- and inter-observer variability inherent to current methods and facilitating the implementation of personalized preventive strategies. In addition, ML models offer greater adaptability to local data structures and can be updated more efficiently than traditional models, improving long-term utility in occupational health systems.

Recent studies have demonstrated the usefulness of ML in the cardiovascular field (36, 37). For example, models based on regularized logistic regression (LR) and random forests (RF) have been used to predict the occurrence of acute coronary syndrome and cerebrovascular events with encouraging results (38–40). More recently, algorithms such as Extreme Gradient Boosting (XGB) have shown superior performance in multiple clinical classification tasks, due to their ability to handle heterogeneous data, robustness to outliers, and computational efficiency (41). Combining these algorithms with widely available low-cost clinical data, such as BMI and glucose, represents an attractive alternative to implement predictive models applicable in occupational settings such as mining.

However, applying ML models to the prediction of CVR still presents important challenges. Among them are the poor adaptation of existing models to populations exposed to extreme working conditions and the limited consideration of the longitudinal evolution of risk factors over time (42). In addition, most previous studies have focused on cohorts of the general population or hospital settings, limiting the generalizability of their results to the reality of mining workers (43). Added to this is the lack of consensus on the best way to operationalize clinically relevant transitions in indicators such as BMI and glucose, key elements for adequate risk stratification in occupational health.

For this reason, improving diagnostic accuracy and interpretative consistency in CVR assessment among mining workers has not only clinical implications, but also economic and social ones, both for workers and for the country. Implementing robust predictive models adapted to the specific needs of this population can optimize available health resources, reduce costs associated with work disability due to cardiovascular events, and ultimately improve quality of life and anticipate mortality among workers. The availability of longitudinal data from occupational health surveillance programs provides a unique opportunity to develop predictive models that integrate the temporal dynamics of risk, enabling more timely and effective preventive interventions (44).

To address these gaps, this study proposes a methodological framework based on ML and longitudinal data to predict clinically relevant transitions in the BMI and BG categories as proxies of increased CVR among high-altitude mining workers. Unlike previous studies that apply ML models to static risk estimation, our work uniquely focuses on predicting longitudinal transitions in risk categories using successive health evaluations. This allows for a more dynamic and proactive approach to risk surveillance. Furthermore, this study is the first to apply this strategy specifically to preemployment occupational surveillance, offering novel information on early metabolic risk changes even before job exposure begins.

This approach aligns with modern occupational health strategies that seek scalable, data-driven solutions to improve the early detection of CVR. The CVR is defined as transitions between the BMI and BG categories detectable on simple and periodic occupational examinations, thus reducing costs and complexity. The framework implements and compares three widely used artificial intelligence algorithms, LR, RF, and XGB, applied to an extensive longitudinal occupational health database. This approach aims to demonstrate that ML models can offer accurate, consistent, and customized predictions for high-risk occupational populations. We hypothesize that ML algorithms, trained in longitudinal occupational health records, can detect early transitions in the BMI and BG categories that signal a rise in CVR, thus supporting timely preventive interventions in high-risk mining workers. This hypothesis stems from the premise that traditional risk scores may not adequately capture dynamic physiological changes in workers facing extreme environments.

In summary, this study:

1. Demonstrates that ML models can accurately predict clinically significant changes in BMI and BG among mining workers using longitudinal occupational health records;
2. Compares the predictive performance of three ML algorithms (LR, RF, and XGB) to identify the most accurate and applicable approach to CVR stratification in this context;
3. Proposes a practical and scalable tool aligned with the Chilean occupational health system to improve early risk detection and preventive care in high-risk populations.

## 2 Materials and methods

### 2.1 Study design and data extraction

A retrospective observational study with an exploratory scope was conducted. The study followed the Strengthening the Reporting of Observational Studies in Epidemiology (STROBE) recommendations (45). All pre-employment examinations of mining workers conducted by a private healthcare provider operating in different regions of the country were included. The sample included the entire population of available records within the study window of 36 months (from the second half of 2021 to the first half of 2024). No probabilistic sampling was performed, and exclusions were only applied to individuals with a single examination, and with missing values or outliers in the variables of interest.

### 2.2 Dataset characteristics

A total of 420,966 pre-employment examination records corresponding to 89,045 unique workers were accessed. This means that each worker often underwent more than one pre-employment examination during the study period and therefore multiple records were available per individual. These repeated evaluations allowed us to construct longitudinal pairs of visits, which were the basis for modeling transitions in the BMI and BG categories over time. In this context, the term “pair” specifically refers to two consecutive examinations by the same worker (for example, exam 1–exam 2, exam 2–exam 3), rather than only the first and last record. Each record was associated with a unique identifier and the date of the examination, allowing information to be grouped by individual and time point. The exam dates for each worker were then chronologically ordered to construct successive pairs of examinations with the aim of modeling individual clinical changes over time.

### 2.3 Data preparation

**Figure 1:**
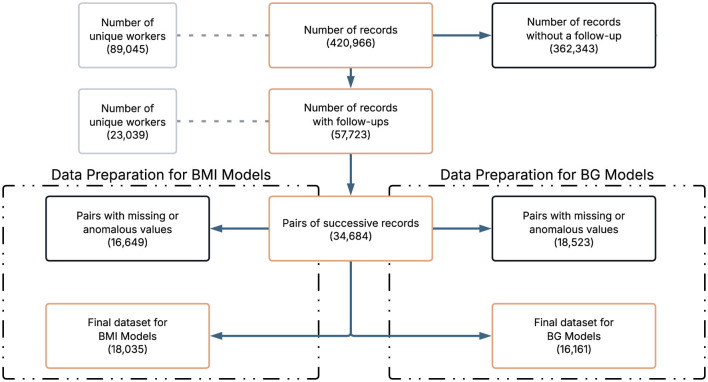
Flowchart for data selection.

Data cleaning and preparation were performed to ensure consistency and analytical quality. Examination record without a follow up were excluded. To ensure comparability of features and facilitate model training, all continuous variables were standardized to follow a standard normal distribution. Any pair of records with missing values (NA) in any of the clinical variables of interest was excluded from further analysis. Although certain ensemble methods, such as Gradient Boosting and Random Forest, are capable of handling missing data internally, we deliberately opt for a complete case strategy. This decision was made to ensure a fair comparison across all model families, including logistic regression, which does not natively accommodate missing values. By applying a uniform approach, we avoided introducing differential sources of bias between architectures. It should be noted that the final sample size of the data set for BMI and BG differ due to data quality and filtering procedures (missing values, outliers, others) applied to each clinical indicator. To ensure fair modeling and avoid data leakage, predictor variables measured at the second visit of each pair were excluded because they could reflect the outcome itself. The only exception was the variable time between tests, which was retained because of its relevance in predicting clinical changes. Figure 1 provides an overview of the data selection process. It is important to note that the construction of longitudinal pairs inherently reduces the number of observations, since for each worker with *N* examination dates, only *N*−1 successive pairs can be formed. This reduction accounts for the transition in Figure 1 from 57,723 records to 34,684 pairs of successive records.

### 2.4 Variables definitions and scenarios

To facilitate clinical interpretation, simplify classification, and align the predictive model with real-world occupational health decision making, the BMI and BG categories were defined based on clinically established thresholds from pre-employment examinations. These categories reflect the levels of health risk considered when assessing a worker's fitness for specific occupational tasks. See Tables 1, 2.

**Table 1:** BMI category definitions.

**Category (code)**	**Definition (kg/m^2^)**
Normal (N)	BMI < 25
Overweight (S)	25 ≤ BMI < 30
Obesity (O)	30 ≤ BMI < 35
Morbid obesity (M)	BMI ≥35

BMI, Body mass index.

**Table 2:** BG category definitions.

**Category (code)**	**Definition (mg/dL)**
Normal (N)	BG < 100
Prediabetic (P)	100 ≤ BG < 125
Diabetic (D)	BG ≥125

BG, Blood glucose.

Given the importance of early detection of health deterioration among workers, the scenarios analyzed were defined as transitions in the BMI and BG categories between two successive medical visits per worker. These scenarios were specifically designed to identify clinically relevant increases in risk, such as progression to obesity or diabetes, that could affect occupational fitness according to preemployment health standards.

**Figure 2:**
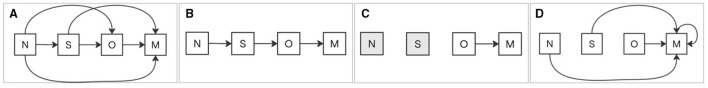
BMI scenarios of category transitions. **(A)** Scenario 1: Any transition to a higher BMI category is labeled positive. **(B)** Scenario 2: Transition to an adjacent higher BMI category is labeled positive. **(C)** Scenario 3: Transition from obesity to morbid obesity is labeled positive. **(D)** Scenario 4: Any transition ending in morbid obesity, regardless of the starting category, is labeled positive. All other cases are labeled negative.

For BMI, the following scenarios were defined: Scenario 1: A transition from a lower category to a higher category of BMI, such as normal to overweight or overweight to obesity, is labeled positive; otherwise, it is labeled negative (Figure 2A). Scenario 2: A transition to an adjacent higher category of BMI, such as normal to overweight but not normal to obesity, is labeled positive; otherwise, it is labeled negative (Figure 2B). Scenario 3: The transition from the obesity category to the morbid obesity category is labeled as positive; otherwise, it is labeled as negative (Figure 2C). Scenario 4: A transition that ends in the morbid obesity category, regardless of the starting category, is labeled as positive; otherwise, it is labeled as negative (Figure 2D).

**Figure 3:**
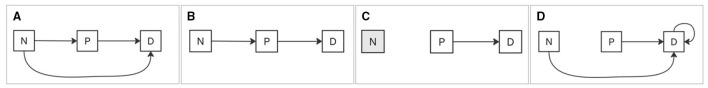
BG scenarios of category transitions. **(A)** Any transition to a higher BG category is labeled positive. **(B)** Transition to an adjacent higher BG category is labeled positive. **(C)** Transition from prediabetic to diabetic is labeled positive. **(D)** Any transition ending in the diabetic category, regardless of the starting category, is labeled positive. All other cases are labeled negative.

Scenario 1: A transition from a lower to a higher category of BG, such as from normal to prediabetic or from prediabetic to diabetic, is labeled as positive; otherwise, it is labeled as negative (Figure 3A). Scenario 2: A transition to a higher adjacent category of BG, such as normal to prediabetic, is labeled positive; otherwise, it is labeled negative (Figure 3B). Scenario 3: The transition from the prediabetic category to the diabetic category is labeled as positive; otherwise it is labeled as negative (Figure 3C). Scenario 4: A transition that ends in the diabetic category, regardless of the initial category, is labeled as positive; otherwise, it is labeled as negative (Figure 3D).

The case distribution is reported in Table 3 for all scenarios.

**Table 3:** Event distribution across scenarios for BMI and BG.

**Scenario**		**1**	**2**	**3**	**4**
BMI	Negative (0)	16,158 (89.6%)	16,170 (89.7%)	5,792 (96.1%)	17,444 (96.7%)
	Positive (1)	1,877 (10.4%)	1,865 (10.3%)	235 (3.9%)	591 (3.3%)
	Total	18,035 (100%)	18,035 (100%)	6,027 (100%)	18,035 (100%)
BG	Negative (0)	13,507 (83.6%)	13,560 (83.9%)	4,192 (97.0%)	15,870 (98.2%)
	Positive (1)	2,654 (16.4%)	2,601 (16.1%)	130 (3.0%)	291 (1.8%)
	Total	16,161 (100%)	16,161 (100%)	4,322 (100%)	16,161 (100%)

BMI, Body mass index; BG, Blood glucose.

### 2.5 Model development and feature selection

To predict each of the defined scenarios, three ML models were implemented: LR, RF, and XGB, as justified below.

**Logistic regression:** The LR was used as a reference model due to its simplicity, interpretability, and widespread validation to predict clinical risk transitions. This parametric method assumes a linear relationship in the logarithmic odds between predictors and outcome probability, which is suitable for modeling the binary transitions targeted in this study, such as a shift to a higher-risk category in BMI or BG between successive visits. This approach enables direct quantification of how each predictor contributes to the probability of health deterioration, facilitating interpretation in a clinical and occupational context. Moreover, its well-established statistical properties make it a useful benchmark against more complex models (46, 47).

**Random forest:** The RF algorithm was selected as a robust nonparametric ensemble method capable of capturing complex, nonlinear interactions between predictors, which are expected when modeling BMI and BG category transitions in a heterogeneous occupational population. This approach identifies complex patterns in risk determinants without imposing strong assumptions on the functional form of variable relationships. In addition, its internal variable importance measure helps identify key factors associated with progression to higher clinical risk levels, helping to interpret and select characteristics in occupational settings. Although alternative models like SVM and deep learning were considered, they were excluded due to interpretability constraints and computational demands in large-scale, real-world applications. The method builds a set of decision trees, each trained on random samples of the dataset, and combines their predictions to minimize overall error (48).

**Extreme gradient boosting:** The XGB algorithm was included because of its ability to effectively model subtle and complex patterns in structured data, such as BMI and BG category transitions over time. This boosting method iteratively corrects previous prediction errors to optimize predictive accuracy, which is particularly useful for detecting gradual, but clinically significant changes that might go unnoticed with linear models. In general, XGB trains sequences of decision trees by minimizing a regularized loss function, enabling high accuracy without overfitting. Its efficient handling of missing and heterogeneous data, together with its competitive performance in clinical prediction tasks, makes it a suitable tool for modeling the individual dynamics of occupational health risk (49).

All variables considered in the initial step are presented in Table 4. The RF was used to rank the variables according to the importance of the characteristics and the most informative predictors were retained for each scenario. Confounders were systematically included in all models to ensure proper adjustment. Collinearity was evaluated at baseline, but was not used as an exclusion criterion, since tree-based algorithms, such as RF, are largely immune to biases caused by collinearity, as supported by previous research (50).

**Table 4:** Sex-based differences in sociodemographic and cardiovascular risk indicators.

	**BG dataset**			**BMI dataset**		
**Biomarker**	**Female (*****n*** **= 611)**	**Male (*****n*** **= 15,550)**	***p*** **-value**	**Female (*****n*** **= 929)**	**Male (*****n*** **= 17,106)**	***p*** **-value**
Age (years)	35.33 ± 9.02	40.09 ± 10.5	< 0.001	37.2 ± 10.95	39.97 ± 10.64	< 0.001
BMI (kg/m^2^)	27.84 ± 4.48	28.62 ± 3.83	< 0.001	28.40 ± 5	28.64 ± 3.97	0.156
Glucose (mg/dL)	88.78 ± 12.82	94.96 ± 16.07	< 0.001	90.72 ± 17.31	94.64 ± 14.94	< 0.001
Triglycerides (mg/dL)	122.25 ± 54.52	150.36 ± 83.17	< 0.001	126.1 ± 52.86	149.75 ± 83.85	< 0.001
Cholesterol (mg/dL)	183.18 ± 28.73	190.09 ± 31.95	< 0.001	184.47 ± 29.89	189.95 ± 31.65	< 0.001
HDL (mg/dL)	53.24 ± 13.74	46.51 ± 9.39	< 0.001	52.6 ± 13.64	46.57 ± 9.39	< 0.001
HR (bpm)	73.32 ± 10.42	70.88 ± 11.13	< 0.001	73.7 ± 10.54	70.91 ± 11.13	< 0.001
Systolic BP (mmHg)	119.98 ± 11.20	125.73 ± 10.17	< 0.001	120.76 ± 12.17	125.73 ± 10.29	< 0.001
Diastolic BP (mmHg)	74.72 ± 9.32	79.43 ± 8.17	< 0.001	75.19 ± 9.84	79.35 ± 8.29	< 0.001
CVR (risk score, 0–1)	0.01 ± 0.004	0.01 ± 0.007	< 0.001	0.01 ± 0.004	0.01 ± 0.007	< 0.001
Hemoglobin (g/dL)	14.8 ± 1.30	15.87 ± 2.11	< 0.001	15.06 ± 1.18	15.89 ± 1.31	< 0.001
Exercise (+)	281 (46.0%)	10,562 (67.9%)	< 0.001	400 (43.1%)	13,999 (81.8%)	< 0.001
Smoking (+)	238 (39.0%)	6,507 (41.8%)	0.167	342 (36.8%)	7,137 (41.7%)	0.003
Alcohol (+)	130 (21.3%)	4,311 (27.7%)	< 0.001	196 (21.1%)	4,753 (27.8%)	< 0.001
Chilean^*^ (+)	561 (91.8%)	14,603 (93.9%)	0.043	803 (86.4%)	16,001 (93.5%)	< 0.001

BMI, Body mass index; HDL, High-Density Lipoprotein; HR, Heart rate; BP, Blood pressure; CVR, Cardiovascular risk; ^*^Chilean: individuals identified with Chilean nationality. *P*-values for categorical variables were obtained using the Chi-square test.

### 2.6 Training procedure and hyperparameter tuning

To detect category transitions in BMI and BG levels, the data set was preprocessed to identify longitudinal changes that crossed clinically defined thresholds. Transition events were encoded as binary outcomes, enabling the use of classification algorithms to predict their occurrence. Special attention was paid to ensuring the temporal consistency of the input variables, aligning baseline and follow-up measurements for each individual. A K-fold repeated cross-validation was implemented to assess model robustness while preserving the longitudinal nature of the data. During cross-validation, feature scaling and encoding were performed within each fold to prevent data leakage. The hyperparameters of the RF and XGB models were optimized using a random grid search strategy, with the objective of maximizing the area under the receiver operating characteristic curve (AUC) while controlling for overfitting. The data set was divided into training subsets (70%) and testing subsets (30%), maintaining the distribution of transition events to reflect the original prevalence, and 95% confidence intervals were estimated for each performance metric. This pipeline enabled models to learn relevant patterns associated with changes in BMI and BG categories, improving predictive accuracy and generalizability (51).

RF relies on bootstrap aggregation, which generates multiple bootstrap samples of the dataset with replacement to train each decision tree. In contrast, the downsampling procedure used to correct the class imbalance during training was performed without replacement, ensuring that the majority class was reduced in a controlled and unbiased manner within each training fold. To address the class imbalance observed in the outcome distribution, several resampling strategies were evaluated, including oversampling, downsampling, and hybrid approaches. The downsampling technique was selected, as the minority class represented only 1.8%–16.4% (depending on the biomarker and scenario) of the observations and, hence, oversampling frequently resulted in severe overfitting. The procedure consisted of random sampling from the majority class within each training fold to match the minority distribution, combined with repeated K-fold cross-validation (K = 7 was used) to ensure robustness and mitigate variability introduced by the sampling process. To further guarantee consistency, we compared results across models using Pearson correlation coefficient. To classify predicted probabilities into binary outcomes, we applied the conventional 0.5 threshold. It is important to note that while the training process was balanced, the final model evaluation, including sensitivity, specificity, and related performance metrics, was conducted on the original imbalanced test set, which preserves the real prevalence of outcomes.

### 2.7 Model evaluation and statistical analysis

Descriptive statistics summarized the overall results of the pre-employment examinations, reporting categorical variables as frequencies and percentages, and quantitative variables as means and standard deviations. Data normality was assessed using the Shapiro-Wilk test. Categorical variables were compared using the Chi-square test, while quantitative variables were compared using Student's t test or the Mann–Whitney U test, depending on data distribution. Pearson correlation coefficient was used to compare the correlation among the outcomes of the different proposed models. Confusion matrices were calculated to derive true positives (TP), false positives (FP), false negatives (FN), and true negatives (TN), reflecting the ability of binary classifiers to correctly identify individuals transitioning to higher-risk BMI or BG categories. The primary evaluation metric was the area under the receiver operating characteristic curve (AUC-ROC), complemented by accuracy, sensitivity, and specificity to provide a comprehensive assessment of model performance.

**Figure 4:**
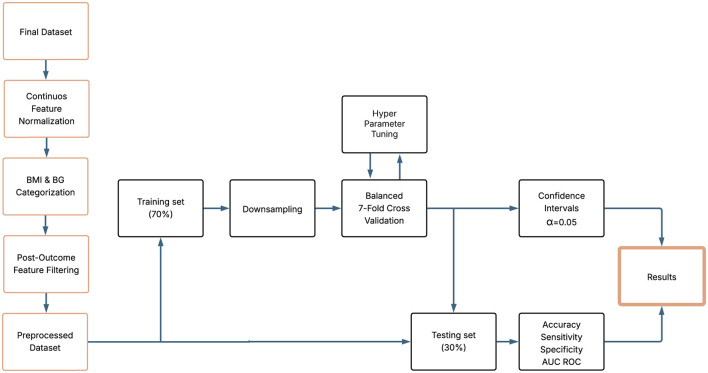
Workflow diagram.

The analysis provided a reliable estimate of the ability of the models to anticipate category transitions and inform early intervention strategies. All analyses and visualizations were performed using the R statistical software package (version 4.1.3). Sampling, model training, and cross-validation were performed using the caret package (version 7.0-1). Data visualization and performance evaluation were performed using pROC (version 1.18.5) and ggplot2 (version 3.5.1). Statistical significance was established at *p* < 0.05. Figure 4 presents an overview of the workflow and variable analysis.

## 3 Results

The study performed a detailed analysis of BMI and BG as primary proxies of CVR, given their critical role in evaluating metabolic health and glycemic control. Other biomarkers and lifestyle factors, such as age, lipid profiles, blood pressure, heart rate, hemoglobin, exercise habits, smoking, alcohol consumption, and nationality, were included solely for sociodemographic characterization and contextual purposes, without in-depth evaluation, to provide a comprehensive background for the primary analysis of BMI and BG. Table 4 shows the description of the data set for each of the biomarkers of interest, BMI and BG.

Tables 5, 6 report the performance of LR, RF, and XGB models in four classification tasks, for BMI and BG, respectively. For each task, the test results are presented alongside cross-validation metrics and their corresponding confidence intervals.

**Table 5:** Model performance metrics for BMI prediction (α = 0.05).

		**Testing**				**7 Fold cross validation**			
**Model**	**Metric**	**1**	**2**	**3**	**4**	**1**	**2**	**3**	**4**
Logistic regression	Accuracy	0.78	0.79	0.81	0.91	0.78 ± 0.03	0.78 ± 0.02	0.77 ± 0.05	0.91 ± 0.02
	Sensitivity	0.81	0.86	0.83	0.95	0.82 ± 0.04	0.82 ± 0.05	0.79 ± 0.06	0.94 ± 0.03
	Specificity	0.76	0.72	0.80	0.86	0.76 ± 0.04	0.74 ± 0.03	0.75 ± 0.07	0.89 ± 0.03
	AUC-ROC	0.87	0.86	0.86	0.97	0.86 ± 0.02	0.86 ± 0.01	0.83 ± 0.06	0.96 ± 0.01
Random forest	Accuracy	0.79	0.79	0.83	0.90	0.79 ± 0.02	0.78 ± 0.01	0.79 ± 0.06	0.91 ± 0.03
	Sensitivity	0.84	0.85	0.88	0.94	0.83 ± 0.02	0.82 ± 0.02	0.82 ± 0.05	0.93 ± 0.02
	Specificity	0.75	0.74	0.78	0.86	0.75 ± 0.02	0.72 ± 0.02	0.74 ± 0.09	0.89 ± 0.06
	AUC-ROC	0.86	0.87	0.85	0.96	0.86 ± 0.01	0.85 ± 0.01	0.85 ± 0.06	0.96 ± 0.02
XGB	Accuracy	0.79	0.78	0.83	0.91	0.80 ± 0.02	0.77 ± 0.02	0.82 ± 0.03	0.92 ± 0.01
	Sensitivity	0.85	0.94	0.93	0.98	0.87 ± 0.03	0.93 ± 0.03	0.94 ± 0.03	0.96 ± 0.02
	Specificity	0.73	0.62	0.72	0.83	0.73 ± 0.04	0.62 ± 0.06	0.70 ± 0.06	0.88 ± 0.03
	AUC-ROC	0.86	0.87	0.84	0.95	0.86 ± 0.02	0.85 ± 0.02	0.86 ± 0.03	0.96 ± 0.02

AUC-ROC, Area Under the Receiver Operating Characteristic Curve; XGB, Extreme Gradient Boosting.

**Table 6:** Model performance metrics for BG prediction (α = 0.05).

		**Testing**				**7 Fold cross validation**			
**Model**	**Metric**	**1**	**2**	**3**	**4**	**1**	**2**	**3**	**4**
Logistic regression	Accuracy	0.69	0.69	0.64	0.74	0.70 ± 0.02	0.69 ± 0.02	0.63 ± 0.05	0.79 ± 0.03
	Sensitivity	0.74	0.77	0.54	0.69	0.77 ± 0.01	0.77 ± 0.03	0.64 ± 0.08	0.76 ± 0.05
	Specificity	0.64	0.62	0.74	0.76	0.63 ± 0.03	0.61 ± 0.04	0.62 ± 0.09	0.83 ± 0.05
	AUC-ROC	0.76	0.77	0.71	0.81	0.77 ± 0.01	0.76 ± 0.02	0.66 ± 0.08	0.87 ± 0.04
Random forest	Accuracy	0.68	0.68	0.68	0.76	0.69 ± 0.02	0.68 ± 0.02	0.72 ± 0.05	0.75 ± 0.06
	Sensitivity	0.72	0.71	0.69	0.70	0.74 ± 0.03	0.73 ± 0.02	0.75 ± 0.11	0.72 ± 0.09
	Specificity	0.64	0.64	0.67	0.83	0.64 ± 0.03	0.62 ± 0.04	0.69 ± 0.07	0.79 ± 0.05
	AUC-ROC	0.75	0.75	0.77	0.83	0.75 ± 0.01	0.74 ± 0.02	0.74 ± 0.07	0.86 ± 0.04
XGB	Accuracy	0.66	0.66	0.65	0.74	0.67 ± 0.01	0.66 ± 0.01	0.64 ± 0.05	0.76 ± 0.06
	Sensitivity	0.68	0.68	0.67	0.76	0.70 ± 0.01	0.68 ± 0.03	0.64 ± 0.09	0.74 ± 0.08
	Specificity	0.65	0.64	0.64	0.72	0.64 ± 0.03	0.63 ± 0.02	0.65 ± 0.10	0.78 ± 0.08
	AUC-ROC	0.72	0.73	0.71	0.83	0.73 ± 0.02	0.72 ± 0.02	0.69 ± 0.09	0.85 ± 0.04

AUC-ROC, Area Under the Receiver Operating Characteristic Curve; XGB, Extreme Gradient Boosting.

**Figure 5:**
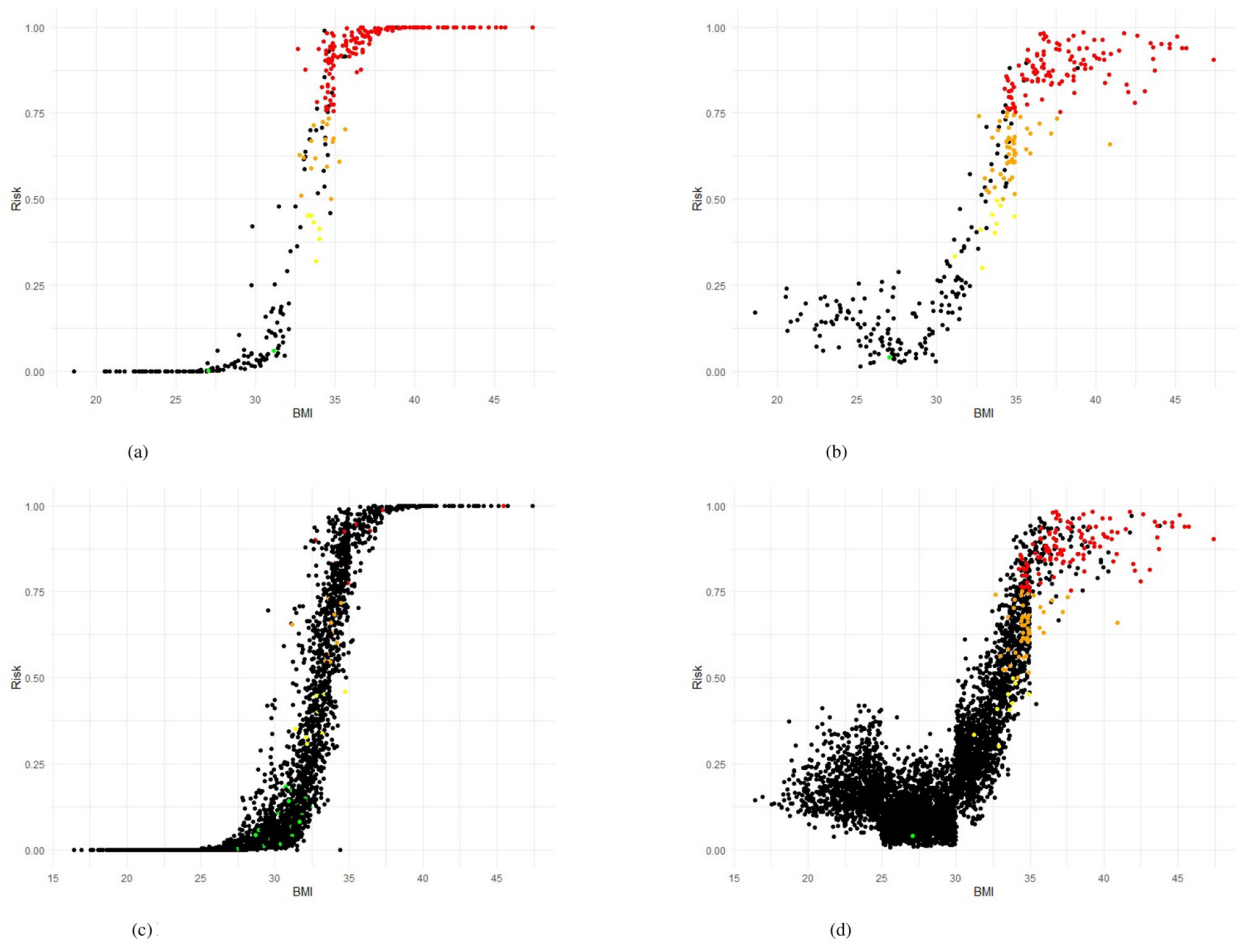
Training and testing sets in Scenario 4 showing Risk BMI for LR and RF models. **(a)** Logistic Regression (LR), training set. **(b)** Random Forest (RF), training set. **(c)** Logistic Regression (LR), testing set. **(d)** Random Forest (RF), testing set.

Table 5 presents the performance of the LR, RF and XGB models in predicting transitions in the BMI categories in four scenarios. All models showed a strong predictive capacity, with XGB performing best in Scenario 4 (increase in morbid obesity, of any origin), achieving an AUC-ROC of 0.95 (95% CI 0.94–0.98) and accuracy of 0.91 (95% CI 0.91–0.93) in the test set. In this scenario, XGB also demonstrated high sensitivity (0.98) and specificity (0.83), indicating excellent detection of transitions to morbid obesity. For Scenarios 1 (any category increase), 2 (one-step category increase), and 3 (increase from obesity to morbid obesity), the models maintained good performance, with AUC-ROC values ranging from 0.84 to 0.87. Scenario 3 showed greater variability, likely due to fewer positive cases (235, Table 3), leading to wider confidence intervals (e.g., LR AUC-ROC: 0.83 ± 0.06). RF slightly outperformed in sensitivity for Scenarios 1 (0.84) and 3 (0.88), while XGB excelled in Scenarios 2 (sensitivity: 0.94). The cross-validation results closely matched the test set outcomes, suggesting minimal overfitting. The high correlation coefficients (0.9016–0.9724, Table 7) confirmed consistent predictions between models, particularly in Scenario 4. As shown in Figure 5, true positives in Scenario 4 were predominantly classified as high or very high risk, with few negative cases misclassified. These results demonstrate the reliability of the models in predicting BMI transitions, especially for severe risk increases, supporting their utility for early intervention in occupational health programs.

**Table 7:** Pearson's correlation coefficients between model predictions for BMI and BG.

**Prediction**	**Comparison**	**1**	**2**	**3**	**4**
BMI	LR vs. RF	0.9016	0.9198	0.9099	0.9630
	RF vs. XGB	0.9517	0.9520	0.9724	0.9525
	XGB vs. LR	0.9256	0.9273	0.9301	0.9696
BG	LR vs. RF	0.8601	0.8753	0.7742	0.9042
	RF vs. XGB	0.8349	0.8461	0.8305	0.9150
	XGB vs. LR	0.7278	0.7362	0.7255	0.8432

BMI, Body mass index; BG, Blood glucose; LR, Logistic regression; RF, Random forest; XGB, Extreme Gradient Boosting.

Table 6 presents the performance of the LR, RF and XGB models in predicting the transitions of the category of BG in four scenarios. All models demonstrated robust predictive performance, with the strongest results in Scenario 4 (increase to diabetic, any origin). Here, RF achieved an AUC-ROC of 0.83 (95% CI: 0.82–0.90) and an accuracy of 0.76 (95% CI: 0.69–0.81) in the test set, with balanced sensitivity (0.70) and specificity (0.83), indicating a reliable detection of transitions to the diabetic category. For Scenarios 1 (any category increase), 2 (one-step category increase), and 3 (increase from prediabetic to diabetic), performance was slightly lower, with AUC-ROC values ranging from 0.71 to 0.77. Scenario 3 showed greater variability, likely due to fewer positive cases (130, Table 3), leading to wider confidence intervals (e.g., LR AUC-ROC: 0.66 ± 0.08 during training cross-validation). LR excelled in sensitivity for Scenario 1 (0.74) and Scenario 2 (0.77), while RF led in Scenario 3 (0.69). The cross-validation results were closely aligned with the results of the test set, confirming minimal overfitting. The correlation coefficients between the prediction of the model (Table 7), ranging from 0.7255 to 0.9150, indicated a strong agreement, particularly in Scenario 4. The models effectively predicted BG transitions, especially for severe risk escalations. Although slightly less robust than BMI predictions, probably due to glucose level variability and lower sample size, these results highlight the potential of the models to support targeted interventions in occupational health settings, even with limited event counts in some scenarios.

To further assess the consistency of the behavior of the model, Table 7 summarizes the pairwise correlations between the predicted probabilities of each model, providing insight into the degree of agreement between the modeling approaches. The correlation analysis revealed strong agreement between the models for the predictions of BMI and BG. These findings confirm that although all models performed well, XGB consistently provided the highest sensitivity and AUC, especially in severe transitions, making it more suitable for early alerts in occupational health. For example, RF and XGB had correlation coefficients greater than 0.95 in all scenarios of BMI. LR was also strongly aligned with both models. This consistency suggests that the choice of model has a limited impact on the predictions, reinforcing both the robustness of the modeling framework and the underlying correlations in the data. The variable importance identified by the RF, XGB, and LR models are shown in the Supplementary Tables 9, 10.

Figures 5a, b present the predicted risk levels vs. BMI for the LR and RF models in Scenario 4, offering a detailed assessment of their ability to classify the transitions to morbid obesity. The scatter plots display risk probabilities (risk identified by the corresponding model) on the y axis, ranging from 0 to 1, plotted against the BMI values on the x axis, with data points colored by predicted risk categories: low risk (< 0.25), yellow for low-mid risk (0.25– < 0.50), orange for mid-high risk (0.50– < 0.75) and high red risk (≥0.75). The black color code indicates individuals who actually did not have the event of interest. Individuals who had a positive event (true positive - TP) are coded by the corresponding risk identified by the model. For both models, the probability of risk demonstrates a consistent increase with higher BMI values, particularly exceeding the BMI of 30, where the majority of TP (red and orange points) are concentrated, indicating an effective identification of people who transition to morbid obesity. The LR plot exhibits a pronounced increase in risk probability around a BMI of 35, with most true positives clustered above a predicted risk of 0.9, suggesting high predictive confidence for severe transitions. Likewise, the RF plot shows a marked elevation beyond the BMI of 30, with a dense aggregation of TP in the high- and very-high-risk zones, corroborating its predictive accuracy. Figures 5c, d also display the predicted risk, but for the test set, as a function of the BMI measured at the most recent examination for the test set for Scenario 4. As noted in the Methods section, the test set is highly imbalanced, with only 3.3% positive cases. This imbalance explains the predominance of black dots in both figures. Similar to the training set plots (Figures 5a, b), an S-shaped distribution can be observed. The Random Forest model provides better discrimination of high-risk workers compared to the Logistic Regression model. This is consistent with Table 5, which shows that sensitivity exceeds specificity across all evaluated models. In the case, for the training set, misclassifications are limited, with few FN occurring in the high-risk range, and the majority of non-events (black points) remaining below a predicted risk of 0.25, reflecting strong discriminative power. This visual evidence aligns with the quantitative results, where the AUC-ROC values reach 0.97 (LR) and 0.96 (RF), Table 5, strengthening the reliability of the models in identifying at-risk individuals based on the previous BMI and supporting their application for early intervention in occupational health settings.

## 4 Discussion

The findings of this study confirm that ML models are accessible and robust tools to predict clinically relevant changes in CVR indicators, such as BMI and BG, among mining workers. Overall, the algorithms implemented demonstrated strong performance, particularly in predicting progression to more severe BMI categories. In particular, the results suggest that BMI changes can be accurately predicted using longitudinal occupational health data, allowing timely preventive interventions. These findings align with previous studies showing that ML models outperform traditional tools. In this research, although this study does not estimate CVR scores directly (e.g., Framingham), it evaluates two key clinical variables that directly relate to CVR. For example, the literature has identified that the presence of diabetes increases the risk of heart failure by 1.87 (1.71 to 2.05) in women and 1.75 (1.59 to 1.93) in men. Similarly, women with a BMI greater than 30, who increase their BMI by 5kg/m^2^, have a risk of heart failure of 1.32 (1.26–1.38), while men have a risk of 1.43 (1.36–1.51) (10).

Consistent with the literature, the high concordance observed between models in BMI predictions reinforces the stability and reliability of this approach, regardless of the algorithm used, and underscores the need for future research to employ larger, more comparable datasets and a broader range of ML models (52). In contrast, glucose predictions were less accurate and showed greater variability, likely due to the more unstable nature of this biomarker and the lower number of positive cases in certain categories, as previously reported in studies on diabetes in occupational settings (53, 54). However, the models performed better in scenarios with more balanced class distributions, and their generalization ability was adequate, as the test results aligned with the cross-validation estimates, indicating no evidence of overfitting (55). The better performance in the prediction of BMI can be explained by the more stable and cumulative nature of this indicator over time, compared to glucose, which tends to fluctuate due to the influence of dietary, metabolic and environmental factors on glycemic variability (56). Previous research has also highlighted the lower reproducibility of glucose as a CVR marker in populations exposed to adverse conditions, supporting our findings (57, 58).

Classical tools for estimating CVR, such as the Framingham and SCORE equations, have shown important limitations, especially when applied in contexts different from those in which they were developed. In particular, they tend to underestimate the actual risk by not taking into account the particularities of the environment and the longitudinal evolution of clinical indicators, as is the case for workers exposed to extreme environments, such as miners, where specific occupational factors are not considered, requiring recent adaptations (59). However, some scores lack validation in external cohorts and others have demonstrated a tendency to miscalculate risk when applied to populations different from their origin relying on classical risk factors, which limit their sensitivity and do not explain all observed cardiovascular events (30). Instead of creating entirely new models, future research should prioritize the adaptation and optimization of current frameworks, focusing on their alignment with occupational cohorts and real-time data acquisition (60). In contrast, the ML models presented in this study overcome these limitations by incorporating temporal data pairs and adapting to the specific characteristics of the population studied, consistent with recent research promoting the use of AI to improve risk stratification in special groups (61, 62).

Another relevant aspect concerns the trade-off between model interpretability and predictive performance. Although complex models such as XGB and RF offer high accuracy and flexibility, they often lack the transparency required for clinical interpretability and trust. Conversely, simpler models such as logistic regression facilitate understanding and decision-making but may fail to capture nonlinear interactions and temporal dynamics. This trade-off has been extensively discussed in occupational health modeling, where stakeholder participation and regulatory compliance often favor interpretable models (63). Therefore, the selection of models should align not only with predictive accuracy, but also with practical implementation constraints in workplace health systems.

However, this study has several limitations. First, using BMI and BG as a proxy of CVR might not be sufficient. However, there are no publicly available data on specific cardiovascular events in individuals belonging to the mining cohort. However, the literature shows that miners and other industrial workers are at increased risk of CVR (64). Another limitation relates to the lack of external validation in other populations. Furthermore, the low prevalence of events in some BG scenarios affected the stability of the model in those categories. Future studies should aim to include a broader range of clinical variables and prospectively validate these models to assess their real-world impact on the prevention of cardiovascular events.

Another methodological aspect concerns the handling of class imbalance. Several approaches were tested, including oversampling and hybrid techniques, but these frequently led to overfitting because of the small size of the minority class. In contrast, downsampling combined with repeated cross-validation yielded more stable and generalizable results, as confirmed by cross-model comparisons (Table 7). This supports the robustness of the chosen strategy, although future research should explore complementary approaches in external datasets. It is also important to note that a classifier may perform better on one class than the other, as sensitivity and specificity measure different aspects of performance, depending on its decision threshold, feature representation, or inherent model bias, even when trained on balanced data. In this context, model outcomes are reported using an unbalanced test dataset, replicating the actual data distribution. The proposed model achieved high AUC-ROC, sensitivity, and specificity. However, the consistently higher sensitivity compared to specificity indicates that the models are more effective at identifying true positive cases, namely workers who are actually at risk.

From a practical point of view, the findings of this study have direct implications for occupational health programs in mining populations. Implementing ML-based predictive models enables early identification of workers at risk of progressing to higher-risk categories, optimizing resources, and reducing the burden of disease and disability in this group. This approach aligns with current trends that promote more dynamic and personalized strategies in occupational health through the use of advanced technologies for the monitoring and follow-up of CVR (65, 66). Therefore, it is highly recommended that these predictive tools be used in conjunction with clinical follow-ups, nutritional interventions, physical activity programs, and periodic health checks to improve the health outcomes of workers.

Finally, this study not only supports the potential of ML as a complementary tool for healthcare teams, strengthening epidemiological surveillance in occupational populations, but also bridges the gap toward more precise, targeted and cost-effective interventions for high-risk workers. Integrating these models into occupational health systems represents a significant advance and a step forward toward more preventive, predictive, and personalized medicine. This reinforces the notion that ML, when contextualized and applied to dynamic occupational data, represents not just a predictive advance, but a paradigm shift in workplace CVR management.

## 5 Conclusion

This study demonstrates that ML models can effectively predict clinically significant changes in BMI and BG levels among mining workers, based on longitudinal occupational health data. Among the models tested, XGB showed particularly strong performance in predicting BMI transitions, with high accuracy and consistent results in different scenarios. These findings underscore the value of incorporating ML-based predictions into occupational health programs to support earlier and more targeted preventive strategies, ultimately improving CVR management in high-risk worker populations by enhancing accuracy and sensitivity as demonstrated in BMI predictions (AUC up to 0.97; sensitivity up to 0.98).

Future research should explore the integration of additional risk factors, such as sleep quality, stress levels, and genetic predispositions, to further refine risk stratification in occupational cohorts. In addition, health authorities could adopt ML-based alert systems to guide targeted preventive actions on a scale. Future studies should aim to externally validate these models in other high-risk occupational settings, such as construction or transportation, where periodic health evaluations and similar physiological stressors are common. Replicating this approach using datasets with longitudinal pre-employment or annual check-up data would provide robust evidence of their generalizability across diverse occupational contexts. Finally, this study provides a scalable evidence-based strategy to improve the early detection of CVR in vulnerable worker populations, aligning with global efforts to reduce the burden of noncommunicable diseases.

## Data Availability

The raw data supporting the conclusions of this article will be made available by the authors, without undue reservation.
