# Supplementary material for: Mining the risk: early cardiovascular detection in workers
Source: Front Med (Lausanne). 2025 Nov 27;12:1678172. doi: 10.3389/fmed.2025.1678172 (PMC12696186; doi:10.3389/fmed.2025.1678172)
Supplement: Supplementary file 1 [file Table_8.pdf]

Table 8: Description of variables used in the analysis, for each successive pair of dates.

**Variable**	**Type**	**Description**
id	Character	Unique patient identifier (Chilean rut)
date_attention	Date	Date of current medical appointment
sex	Categorical	Patient's gender (male/female/other)
health_coverage	Categorical	Healthcare provider (fonasa/isapre)
age_range	Categorical	Patient's age range (e.g., 41–55 years)
nationality	Categorical	Patient's country of origin
BMI	Numeric	Current BMI (kg/m^2)
glycemia	Numeric	Current BG level (mg/dL)
cholesterol	Numeric	Current total cholesterol level (mg/dL)
creatinine	Numeric	Current creatinine level (mg/dL)
hemoglobin	Numeric	Current hemoglobin level (g/dL)
triglycerides	Numeric	Current triglycerides level (mg/dL)
name_region	Categorical	Patient's municipality of residence
bpressure_sisto	Numeric	Current systolic blood pressure (mmHg)
prev_BMI	Numeric	BMI from previous appointment (kg/m^2)
prev_glycemia	Numeric	Glucose from previous appointment (mg/dL)
prev_cholesterol	Numeric	Cholesterol from previous appointment (mg/dL)
prev_creatinine	Numeric	Creatinine from previous appointment (mg/dL)
prev_hemoglobin	Numeric	Hemoglobin from previous appointment (g/dL)
prev_triglycerides	Numeric	Triglycerides from previous appointment (mg/dL)
prev_bpressure_sisto	Numeric	Systolic BP from previous appointment (mmHg)
prev_date	Date	Date of previous medical appointment
time_between_tests	Numeric	Days between current and previous appointment
cat_f_BMI	Categorical	Current BMI category (e.g., normal/overweight)
cat_f_glycemia	Categorical	Current glucose category (e.g., normal/high)
cat_i_BMI	Categorical	Previous BMI category (e.g., normal/overweight)
cat_i_glycemia	Categorical	Previous glucose category (e.g., normal/high)

BMI = Body Mass Index; BG = Blood Glucose; BP = Blood Pressure. Variables prefixed with *prev_* correspond to measurements from the previous medical appointment. Variables prefixed with *cat_f_* represent categorical classifications at the final appointment, and variables prefixed with *cat_i_* represent categorical classifications at the initial (previous) appointment.
