# Supplementary material for: Mining the risk: early cardiovascular detection in workers
Source: Front Med (Lausanne). 2025 Nov 27;12:1678172. doi: 10.3389/fmed.2025.1678172 (PMC12696186; doi:10.3389/fmed.2025.1678172)
Supplement: Supplementary file 2 [file Table_9.pdf]

Table 9: Variable importance across BMI prediction models and their average.

	RF				LR				XGB				
**Variable**	**1**	**2**	**3**	**4**	**1**	**2**	**3**	**4**	**1**	**2**	**3**	**4**	$\bar{X}$
prev_BMI	100	100	100	100	89.14	90.67	100	100	100	100	100	100	98.32
cat_i_BMI_overweight	7.76	7.67	NA	27.29	96.19	93.74	NA	0.02	0	0	NA	0	25.85
cat_i_BMI_obese	8.2	8.42	NA	7.23	100	100	NA	0	0	0	NA	0	24.87
time_between_tests	15.42	14.62	13.68	7.81	17.19	9.42	19.23	17.21	4.6	2.35	6.51	0.07	10.68
age_range_41_55	NA	NA	NA	NA	0	0.89	20.28	10.85	NA	NA	NA	NA	8.01
prev_bpressure_sisto	8.69	9.66	7.91	9.82	4.78	0	10.21	19.14	0.38	0.9	1.89	0.18	6.13
prev_triglycerides	7.34	8.85	5.08	8.07	4.76	4.93	15.03	13.43	0.67	1.21	1.3	0.97	5.97
age_range_over_55	NA	NA	NA	NA	7.69	3.63	6.85	3.68	NA	NA	NA	NA	5.46
prev_hemoglobin	5.69	6.9	4.2	6.21	2.63	2.99	17.98	15.34	0.28	1.39	0.71	0.59	5.41
prev_glycemia	9.36	10.41	6.96	10.11	4.79	3.26	9.51	6.3	0.79	0.88	0.77	0.49	5.30
cat_i_BMI_morbid	3.24	2.03	NA	39.81	0.1	0.25	NA	0	0	0	NA	0	5.05
prev_cholesterol	7.75	7.87	4.54	6.28	9.61	7.18	10.8	1.88	1.3	0.63	1	0.07	4.91
edu_basic	4.67	4.87	5.24	5.6	3.85	9.95	10.39	11.32	0.34	0.54	0.01	0.13	4.74
edu_technical	5.48	5.23	5.79	5.41	1.07	10.09	15.68	1.15	0.32	0.55	2.96	0.64	4.53
sex_male	0.94	0	0.86	2.12	1.23	3.55	17.89	25.8	0.11	0.09	0.4	0.13	4.43
name_region	2.38	1.64	1.58	4.56	3.59	10.23	8.09	17.4	0.06	0.21	0	0	4.15
edu_professional	4.97	4.02	2.92	5.02	6.43	5.64	8.45	9.9	0.69	0.35	0	0.18	4.05
edu_total	5.05	4.13	4.2	4.88	3.96	7.79	12.15	4.35	0.36	0.33	0.02	0.22	3.95
prev_creatinine	8.03	8.4	8.43	6.55	5.39	0.94	0.52	2.17	0.92	1.92	3.01	0.22	3.88
nationality_other	0.85	0.3	1.05	0	0.31	8.8	21.4	11.32	0	0.21	0.52	0	3.73
n_records	3.5	3.43	2.76	4.69	8.64	14.02	0	6.65	0.15	0.55	0	0	3.70
occupation_professional	2.84	2.25	1.93	4.92	8.94	1.95	2.51	17.71	0.08	0.1	0	0.01	3.60
occupation_basic	2.97	2.06	2.04	4.37	7.17	3.85	2.78	17.54	0	0.2	0	0	3.58
total	2.54	1.81	1.51	4.31	7.77	2.36	4.71	14.34	0.11	0.02	0	0	3.29
p_indicator	6.54	5.06	3.92	5.3	2.86	4.8	0.33	8.41	0.87	0.65	0	0.36	3.26
indigenous_percentage	5.56	5.5	5.24	5.44	2.16	0.34	0.04	10.13	0.73	1.29	0.79	0.24	3.12
occupation_technical	2.76	2.11	1.66	4.48	0.26	9.64	2.55	11.31	0.29	0.07	0	0	2.93
cat_f_glycemia_pre_diabetes	0.84	NA	0.06	NA	4.59	NA	11.09	NA	0	NA	0	NA	2.76
indigenous	4.1	3.37	2.97	5.11	1.82	7.41	6.36	0.03	0.44	0.25	0	0.23	2.67
cat_f_triglycerides_normal	NA	NA	0.08	NA	NA	NA	7.22	NA	NA	NA	0	NA	2.43
cat_f_bpressure_sisto_high	0.87	NA	0.33	NA	4.58	NA	7.64	NA	0	NA	0	NA	2.24
cat_f_glycemia_diabetes	0.23	NA	0	NA	3.32	NA	7.8	NA	0	NA	0	NA	1.89
health_coverage_isapre	0.89	0.51	0.01	0.7	0.76	9.97	1.06	4.17	0	0.13	0	0	1.52
cat_f_triglycerides_high	0.46	NA	NA	NA	3.49	NA	NA	NA	0	NA	NA	NA	1.32
cat_f_triglycerides_borderline	0.83	NA	0.42	NA	4.6	NA	1.69	NA	0.08	NA	0	NA	1.27
age_range_41_55	1.25	0.81	1.17	1.11	NA	NA	NA	NA	0	0.03	0	0	0.55
age_range_over_55	1.18	0.05	0.35	0.74	NA	NA	NA	NA	0.22	0.04	0	0	0.32
cat_f_triglycerides_very_high	0	NA	NA	NA	NA	NA	NA	NA	0	NA	NA	NA	0

RF = Random Forest; LR = Logistic Regression; XGB = Extreme Gradient Boosting. Variables prefixed with *prev_* correspond to biomarker measurements from the previous appointment. Variables prefixed with *cat_* represent categorical transformations of continuous variables (e.g., normal, high, borderline). Socio-demographic predictors include: *sex_male* (male sex), *age_range_41_55*, *age_range_over_55*, *nationality_other*, and *name_region* (region of residence). Socioeconomic variables include: *p* (proportion of population), *p_indigenous* (proportion of indigenous population), *p_edu_total*, *p_edu_basic*, *p_edu_technical*, *p_edu_professional* (proportions of education levels), *professional_count*, *basic_count*, *technical_count*, and *total_count* (counts of educational attainment). *health_coverage_isapre* indicates private health insurance coverage. Clinical categorical variables include: *cat_i_glycemia_prediabetes*, *cat_i_glycemia_diabetes*, *cat_bpressure_sisto_high* (systolic BP high), and triglyceride categories (*cat_triglycerides_normal*, *borderline*, *high*, *very_high*). *n_obs* corresponds to the number of available observations.
