# Supplementary material for: Mining the risk: early cardiovascular detection in workers
Source: Front Med (Lausanne). 2025 Nov 27;12:1678172. doi: 10.3389/fmed.2025.1678172 (PMC12696186; doi:10.3389/fmed.2025.1678172)
Supplement: Supplementary file 3 [file Table_10.pdf]

Table 10: Variable importance across BG prediction models and their average.

	RF				LR				XGB				
**Variable**	**1**	**2**	**3**	**4**	**1**	**2**	**3**	**4**	**1**	**2**	**3**	**4**	$\bar{X}$
prev_glycemia	100	100	100	100	62.6	54.19	100	100	100	100	100	100	93.07
time_between_tests	53.42	55.49	28.55	13.06	34.25	33.81	35.83	58.73	44.65	43.2	34.04	17.55	37.72
age_range_41_55	NA	NA	NA	NA	13.68	15.7	45.95	55.57	NA	NA	NA	NA	32.73
prev_BMI	48.3	48.88	28.9	13.49	20.44	16.97	0.07	52.83	43.97	43.36	21.58	11.99	29.23
age_range_over_55	NA	NA	NA	NA	19.44	18.02	40.15	38.46	NA	NA	NA	NA	29.02
cat_i_glycemia_prediabetes	19.85	23.87	NA	0.38	100	100	NA	10.68	0	0	NA	0.06	28.32
prev_hemoglobin	23.3	24.97	21.53	5.01	5.12	7.88	13.08	15.43	16.01	18.87	22.14	2.47	14.65
prev_triglycerides	26.17	27.27	11.07	5.5	5.41	1.87	27.74	3.59	22.97	23.7	10.38	2.87	14.05
prev_cholesterol	27.21	30.82	10.84	8.03	6.62	4.92	7.43	9.42	22.04	25.83	2.74	6.69	13.55
prev_creatinine	27.24	31.34	20.68	6.7	0.62	8.45	0.78	5.29	21.02	22.7	10.63	6.89	13.53
p	15.29	15.68	11.12	6.21	4.67	0.5	10.25	48.5	12.62	13.08	9.39	7.24	12.88
p_indigenous	16.33	16.61	16.08	6.2	4.98	1.48	11.49	31.46	12.47	12.12	10.72	3.24	11.93
p_edu_total	16.13	15.91	16.77	4.07	8.05	5.53	2.18	26.39	14.08	12.33	17.24	3.72	11.87
p_edu_basic	15.74	17.11	11.86	5.04	10.84	7.68	3.72	23.17	13.23	13.59	9.64	5.68	11.44
p_edu_professional	12.64	13.06	9.94	3.89	8.93	7.43	13.97	29.31	9.95	9.21	2.24	5.59	10.51
p_edu_technical	15.71	16.11	16.85	5.49	10.69	6.29	2.75	3.72	13.29	11.2	11.77	4.89	9.90
professional_count	8.6	9.39	8.59	1.93	2.89	2.51	32.97	29.9	5.69	4.6	3.83	3.91	9.57
basic_count	7.84	8.99	9.7	1.62	4.88	3.39	29.9	23.64	4.83	5.68	4.33	1.3	8.84
total_count	7.04	7.97	9.29	1.2	4.87	2.57	26.64	22.72	4.56	5.76	7.57	1.78	8.50
indigenous	11.8	12.5	11.96	2.6	4.5	2.66	9.55	15.02	7.71	11.19	2.79	2.08	7.86
n_obs	10.49	11.56	12.55	3.04	2.64	5.15	5.83	1.07	7.92	10.63	13.81	3.48	7.35
health_coverage_isapre	4.29	5.82	2.97	0.65	11.03	14.68	10.98	25.08	2.78	3.71	2.31	0.75	7.09
cat_bpressure_sisto_high	5	6.18	3.48	0.83	0.84	9.05	28.61	0	3.25	5.39	3.19	0.78	5.55
edad_range41-55	4.68	5.84	4.83	2	NA	NA	NA	NA	3.46	2.98	4.2	3.49	3.94
sex_male	1.95	3.1	0.97	0	12.44	16.55	1.46	6.28	1.3	2.08	0	0	3.84
technical_count	7.25	8.71	6.72	1.53	2.76	0.1	4.3	0.35	3.48	4.99	3.49	2.23	3.83
cat_triglycerides_borderline	2.49	2.58	2.02	0.27	3.21	7.91	21.69	0.71	1.27	0.68	0.99	0	3.65
name_region	7.48	8.49	6.9	1.46	5.22	0	1.76	2.33	2.01	2.42	2.26	1.67	3.5
edad_range>55	3.74	4.09	7.32	0.76	NA	NA	NA	NA	1.81	1.99	7.9	0.3	3.49
cat_triglycerides_normal	NA	1.98	1.07	0.54	NA	0.3	25.06	1.12	NA	0.05	0	0	3.35
nationality_other	NA	2.56	1.05	0.58	NA	3.97	6.9	6.75	NA	0.82	0	0	2.51
cat_i_glycemia_diabetes	2.49	2.91	NA	0.01	0	0.5	NA	14.25	0	0	NA	0	2.24
cat_triglycerides_very_high	0	0	0	0.22	2.3	5.15	0	17.75	0	0	0	0	2.12
cat_triglycerides_high	1.67	NA	NA	NA	2.58	NA	NA	NA	0	NA	NA	NA	1.42

RF = Random Forest; LR = Logistic Regression; XGB = Extreme Gradient Boosting. Variables prefixed with *prev_* correspond to biomarker measurements from the previous appointment. Variables prefixed with *cat_* represent categorical transformations of continuous variables (e.g., normal, high, borderline). Age predictors include *age_range_41_55*, *age_range_over_55*, *edad_range41-55*, and *edad_range>55*. Socio-demographic predictors include *sex_male*, *nationality_other*, *name_region* (region of residence), and *indigenous* (self-identified indigenous status). Socioeconomic variables include *p* (population proportion), *p_indigenous* (proportion of indigenous population), *p_edu_total*, *p_edu_basic*, *p_edu_technical*, *p_edu_professional* (proportions of education levels), *professional_count*, *basic_count*, *technical_count*, and *total_count* (counts of educational attainment). *health_coverage_isapre* indicates private health insurance coverage. Clinical categorical variables include *cat_i_glycemia_prediabetes*, *cat_i_glycemia_diabetes*, *cat_bpressure_sisto_high* (systolic BP high), and triglyceride categories (*cat_triglycerides_normal*, *borderline*, *high*, *very_high*). *time_between_tests* = days between successive appointments; *n_obs* = number of available observations.
